# Relationship between Driving Ability and Physical Fitness Factors in Older Adults: A Multiple Linear Regression Analysis

**DOI:** 10.3390/ijerph21060660

**Published:** 2024-05-22

**Authors:** Akihiko Katayama, Takenori Abe, Ayako Hase, Nobuyuki Miyatake

**Affiliations:** 1 Faculty of Sociology, Shikoku Gakuin University, Zentsuji-shi 765-8505, Kagawa, Japan; 2 Promoting Exercise Association in Kagawa, Marugame-shi 763-0074, Kagawa, Japan; t.abe@a-kao.jp; 3 Department of Clinical Psychology, Faculty of Medicine, Kagawa University, Kita-gun 761-0793, Kagawa, Japan; hase.ayako@kagawa-u.ac.jp; 4 Department of Hygiene, Faculty of Medicine, Kagawa University, Kita-gun 761-0793, Kagawa, Japan; miyatake.nobuyuki@kagawa-u.ac.jp

**Keywords:** driving ability, physical fitness factors, older adults

## Abstract

The number of older drivers is increasing with the aging population; this has led to concerns about traffic accidents involving older drivers. For older adults, the automobile is not just a means of transportation, but a life necessity that promotes social activities and maintains and improves health-related quality of life. In this study, we aimed to clarify the relationship between driving ability and physical fitness factors among 70 older adult drivers using a single regression analysis and multiple regression models adjusted for age, sex, and other factors. Driving ability was evaluated by driving an actual car on an ordinary road without a simulator. The single regression analysis revealed no relationship between driving ability and any physical fitness factor. In the multiple regression model analysis, only grip strength was an important explanatory factor; however, the driving ability scores decreased as grip strength scores increased. By clarifying the physical fitness factors that influence the maintenance and improvement of driving ability, it is possible to propose more efficient intervention programs to maintain and improve driving ability. We could not identify the relevant physical fitness factors in this study; therefore, further research is required to improve safe driving among older adults.

## 1. Introduction

Based on the current global demographic situation, the percentage of the world population aged ≥ 65 years was projected to increase from 10% in 2022 to 16% in 2050 [1,2]. With the increase in the number of older adults worldwide, countries and regions with aging populations need to take various actions, including reforming welfare policies, strengthening health promotion measures, improving the living environment, and implementing employment promotion measures in the labor system [3,4]. With the aging population, the number of older adult drivers is increasing [5,6]. Consequently, traffic accidents involving older adult drivers are increasing, which has become a global concern [7]. Older adults experience difficulty driving a car because of age-related changes in physical functions [8,9].

This global trend has also been observed in Japan, where a particular feature article on “Emergency Traffic Safety Measures for Elderly Drivers” was published in the White Paper on Traffic Safety issued by the Japanese government in 2020 [10]. In Japan, a system has been introduced that allows older adult drivers to voluntarily return their driver’s licenses (license revocation upon request) [11]. This is a measure to reduce the number of automobile accidents caused by older adult drivers who feel uneasy about driving because of the aging-induced decline in their physical functions. In addition, in Japan, a driving test was introduced in May 2022 for drivers aged ≥75 years at the time of license renewal and who had committed certain traffic violations within the past three years, and a driving ability test using a real car was implemented [12].

While traffic accident countermeasures for older adult drivers are currently being implemented, automobile driving is very important to older adults [13,14,15]. The use of automobiles is essential for the independent living of older adults, particularly in depopulated areas where public transportation, such as trains and buses, is scarce [16,17]. Driving a car and getting around on one’s initiative is not only a matter of physical transportation but also a life necessity for community-dwelling older adults to live within society. It has been reported that maintaining the ability to drive a car safely and move at will is also associated with maintaining health-related quality of life [18,19].

Research and development of safe vehicles with specifications for older adult drivers is ongoing [20,21,22], indicating an “automotive development approach” to developing safe vehicles. Automated vehicle development is progressing rapidly; however, it will take a while before fully automated vehicles are delivered to the end driver and all regions [23]. Furthermore, it is a fact that, in all cases, the driver operates the vehicle. For safe driving, it is essential to approach older adult drivers to ensure they maintain their driving abilities. Therefore, the “approach to the driver” is a critical perspective.

Factors associated with driving ability, sensory function, cognitive function, and motor function are necessary and interact with each other. Regarding motor function in older adult drivers, providing and practicing a healthy exercise program may maintain and improve their ability to turn [24], indicating that an approach to maintain and improve the physical function of older adults can maintain and improve their driving ability. Therefore, healthy exercise and physical activity practices can be one of the methodologies for improving the maintenance of driving ability. However, which physical fitness factors are associated with the driving ability of older adults or individual physical fitness index factors remain unclear. By clarifying the important physical fitness factors determining driving ability, developing a more efficient methodology for maintaining and improving driving ability through healthy exercise practices is possible. In the present study, we aimed to clarify the relationship between driving ability and physical fitness factors among community-dwelling older adults whose driving abilities were expected to decline.

## 2. Materials and Methods

### 2.1. Participants’ Recruitment

We obtained the cooperation of local municipal administrative agencies, community centers, and federations of senior citizen clubs in a region to recruit participants. At each facility, flyers advertising the recruitment of participants were distributed, and meetings were held to explain the research content. In addition, flyers advertising the recruitment of participants were distributed as inserts in local newspapers. Then, requests for participation were accepted by mail and telephone for one week.

The recruitment criteria were as follows: (1) a regular driver’s license, (2) daily driving, “driving a motor vehicle as a daily activity, such as shopping, at least once a week”, and (3) recent anxiety about driving. Because older adults were targeted in the present study, the age requirement was ≥65 years. Regarding their health and physical conditions, the participants had no contraindication to exercise based on a doctor’s diagnosis and could get to the venue of the health class without assistance from others. When recruiting participants, the number of participants in each category was not restricted based on age, sex, or region of residence.

An explanatory meeting was held with those who wished to participate in the study. We ensured they were accurately informed of the study purpose, details, research schedule, and other information. Seventy-five participants attended the briefing session and provided written informed consent to participate after understanding the study’s objectives. Notably, participation in the study was entirely voluntary, and all 75 participants expressed their willingness to participate.

### 2.2. Study Design

In this study, we used a cross-sectional design and conducted two measurement sessions. With the cooperation of the driving school, participants’ driving ability was assessed on a public road outside the driving school. Participants’ physical fitness factors were assessed by a qualified health and exercise instructor at a community gymnasium.

First, the relationship between the participants’ driving ability and physical fitness factors was analyzed using a single regression analysis. Multiple regression analysis was performed using participants’ driving ability as the objective variable and their attributes and physical fitness factors as the explanatory variables.

### 2.3. Determination of the Number of Participants

This study was a secondary analysis of data obtained from a previous study on improving the driving ability of community-dwelling older adults [25]. The number of participants recruited was determined based on the sample size calculations of the randomized controlled trial in the previous study. The expected dropout rate of participants during the study period was calculated as 10%. As a result, 74 participants were needed for this study, and participants were recruited based on this calculation.

### 2.4. Measurement Items and Evaluation Indicators

#### 2.4.1. Basic Attributes

Physical fitness factors were assessed using a self-administered questionnaire to provide information on participants’ essential attributes. Height and weight measurements were performed using regular equipment during the physical fitness testing. Each participant’s body mass index (BMI) was calculated as weight (kg)/height (m)^2^.

#### 2.4.2. Driving Ability Measurement

Previous studies on automobile driving abilities often used driving simulators to measure driving abilities [26,27]. However, license holders undoubtedly drive automobiles on public roads daily.

Under the laws in force in Japan, a certified driving skill test operator conducts a driving proficiency test for a motor vehicle before a driver’s license is issued. These skill test operators have been issued a skill test operator qualification certificate after passing a training course and a qualification examination conducted by the Japan Public Safety Commission under the Road Traffic Law of Japan (Article 99-2, Paragraph 4). The examinations are conducted not only on school courses in designated driving schools but also on “public roads”, which are general roads known as examination courses. Regarding the implementation of driving tests at such sites, driving ability measurements were evaluated in the present study using data obtained from skill test operators’ evaluations of driving ability rather than assessing driving abilities using a driving simulator.

One concern regarding the evaluation of driving ability by driving skill test operators is the difficulty of unifying the evaluation level regarding measurement values or calibration. This concern was thoroughly discussed among the staff members in charge of the study, the person in charge of the measurement, and the driving skill test takers. The following measures were taken to implement the measurements in the present study: (1) all driving skill test operators in charge of measurement and evaluation were qualified according to Japanese law; (2) regular training on testing techniques was conducted; and (3) prior to measurement, the scoring criteria were reconfirmed to ensure that the evaluation criteria were leveled among the skill test operators. The data quality was sufficient for research purposes.

Driving ability measurements were conducted for each participant at a cooperative driving school. The health condition of the participant was verified; the measurement schedule was explained; the measurement course to be covered was explained; and the vehicle to be used was explained in a series of steps. After confirming their willingness to participate in the study, the examiner in charge conducted measurements on public roads. After the measurements were completed, participants’ health condition was checked. The measurement time per participant was approximately 50 min. Driving ability was measured after the participant drove the vehicle for approximately 20 min on a designated public road course, with the skill test operator riding in the passenger seat of the vehicle. Measurements were conducted using a vehicle owned by a driving school. The same public road course was used for all participants. The course was set up according to the course used during the automobile license examination, starting and ending at the driving school. It was a circular course around a nearby urban area. The course included signals, stop signs, right turns, left turns, and all items required for the automobile license test. Highways were not included. The driving ability evaluation was conducted using the automobile license test administered in Japan. Points were added if the driver failed to check the surrounding conditions or disregarded the stop sign. Therefore, the higher the number of points at the end of the measurement, the lower the driving ability level.

Because the measurements were conducted on public roads, safety was prioritized in all aspects. The skill test operators who conducted the measurements agreed that if any dangerous driving was detected during the measurement, the vehicle would be stopped immediately, and the measurement would be discontinued. In such cases, data from participants whose measurements were interrupted were excluded from the analysis without remeasurement. Note that this study had no interruptions in the middle of the measurement.

Furthermore, we described the measurement elements of driving ability. When a person drives a car, the driving behavior consists of three elements: “cognition”, “judgment”, and “operation [26]”. Regarding “cognition”, it involves understanding the external environment factors through senses such as sight and hearing. It indicates the ability of the driver to check traffic signs, signals, and other conditions around the vehicle and recognize abnormalities and hazards. Regarding “judgment”, it represents the driver’s own decision. It indicates the ability of the driver to analyze traffic information and decide how the vehicle should be driven using the results of the external environment confirmed through perception. During judgment, the driver decides how to act as a driver, such as stopping the car, going straight, turning right, or turning left. Regarding “operation”, it is the actual execution of the diver’s action according to the “decision” made by the driver. Specific examples of driver actions include steering the vehicle, braking, and acceleration.

In the present study, the driving ability was evaluated based on the “cognition”, “judgment”, and “operation” phases. In each phase, a one-point number was added when a point reduction item on the driving ability test was identified. In daily driving, the driver’s behavior is continuous as a sequence, and each of the “cognition”, “judgment”, and “operation” phases continuously affects the driver’s behavior. Therefore, in addition to the individual score evaluation at each stage, three categories of composite items under continuous conditions were also included in the analysis: “cognition” + “judgment” score evaluation, “judgment” + “operation” score evaluation, and “cognition” + “judgment” + “operation” score evaluation.

In this study, the analysis is being conducted from the perspective of its relevance to the physical fitness factor. In the above three elements, “judgment” and “operation” were selected as items mainly related to the physical fitness element. In addition, since driving a car is performed under continuous conditions, the categories of “judgment” + “operation” were created and set as the objective variables in the correlation analysis items and in the stage of conducting multiple regression analysis.

#### 2.4.3. Basic Physical Fitness Measurement

Physical fitness was measured as a factor associated with driving ability. In the present study, the physical fitness component items were measured at a local gymnasium by staff members who were health and exercise experts. The measurement items of the new physical fitness test by the Ministry of Education, Culture, Sports, Science, and Technology, which is currently widely used in Japan, were adopted in the present study [28,29]. The items measured were whole-body muscle strength index using grip strength, flexibility index using long-body forward bending, balanced index using standing on one leg with eyes open, and mobility index using walking over a 10 m obstacle. Grip strength was measured by a grip strength meter, and the average value of the right and left hands was used as the grip strength measurement data. For the long-body forward bending measurement, the subjects sat on the floor with their legs extended and stretched their hands together in the direction of their toes, and the maximum value was used as the data for the long-body forward bending measurement. In the one-leg stand with open eyes measurement, the subject shifted to the one-leg stand position by lifting the left or right foot off the floor at the start cue, and the time until the one-leg stand posture was broken was measured and used as the data for the one-leg stand with open eyes measurement. For the 10 m obstacle walking measurement, the time to move a distance of 10 m with an obstacle placed was measured by walking and was used as the 10 m obstacle walking data.

Lower limb muscle strength measurements were conducted considering the stepping motion with the legs, which is unique to the driving motion. A 30 s chair stand test was performed to measure the endurance of lower limb muscle strength [30,31]. A five-time chair standing test was performed to measure the instantaneous strength of the lower limb muscles [32,33]. The Timed Up and Go (TUG) test is a comprehensive evaluation index that includes walking ability [34,35] and has been used in many care settings for community-dwelling older adults as a comprehensive test to determine gait ability, dynamic balance, and agility. This test is also considered helpful in determining the risk of falling. It is highly reliable for assessing motor function in older adults, and many research reports exist. This test was adopted in the present study as a comprehensive physical fitness index for older adults, and measurements were conducted.

### 2.5. Measurement of Executive Function and Alertness Function

The Trail Making Test (TMT) (Japanese version of the TMT [TMT-J]) was used as a measure of executive and alertness functions in older adults [36,37,38]. The TMT, which has been used in Japan as a driving aptitude test for older adults under certain circumstances, is an internationally used measure of executive function for all age groups, and its high reliability and validity as a measure of attentional function have been widely reported. It can comprehensively determine attentional function, working memory, spatial cognition, and processing speed quickly.

The TMT comprises two tests: part A (TMT-A) and part B (TMT-B). The TMT-A consists of only numbers. The participants were asked to connect the numbers randomly written on the paper from “1” to “25” in sequence by drawing a line with a pencil, and the time (in seconds) taken to complete the sequence was measured. The TMT-B consists of numbers from “1” to “13” and hiragana (like the English alphabet A, B, C, etc.) from “A” to “L”. The numbers and hiragana were alternately connected in the same way, and the completion time (in seconds) was measured. The participants underwent a practice test before the main test. They were instructed to complete the test as quickly as possible and to keep their pencils on paper.

The shorter the time required to complete the TMT, the better.

### 2.6. Analytical Methods and Statistical Processing

In the present study, measurements were presented as mean (standard deviation) for continuous variables and as number of persons (percentage, %) for categorical variables. Descriptive statistics were computed for all the variables. The index involving the combination of “judgment” and “operation”, which were highly relevant as physical fitness factors, was used as the driving ability index in this study. The relationship between the driving ability index and physical fitness factors was analyzed using a single regression analysis. In addition, a multiple regression model was generated and used to determine the relationship between driving ability and physical fitness factors, adjusting for age, sex, and other attributes of the participants.

Statistical analysis was performed using Excel statistics Version 4.06 (Bell Curve for Excel; Social Survey Research Information Co., Tokyo, Japan). Statistical significance was set at *p* < 0.05 (two-tailed).

### 2.7. Ethics

The study was conducted according to the guidelines of the Declaration of Helsinki. This study was approved by the Ethical Committee of Shikoku Gakuin University, Zentsuji City, Kagawa Prefecture, Japan (approval number: 2021001). This protocol was registered with the University Hospital Medical Information Network Clinical Trials Registry (registration number: UMIN000044706).

## 3. Results

### 3.1. Participants’ Characteristics

After an explanatory meeting, all 75 participants agreed to participate in the study. However, during the physical fitness and driving ability measurements, five people declined to participate owing to family objections to their participation or their physical condition, and 70 persons were included in the final analysis.

Table 1 presents the characteristics of the 70 participants included in the final analysis. The mean age of the participants was 74.9 years (standard deviation: 3.8 years), and 54 patients (77.1%) were female (Table 1).

### 3.2. Correlations

The association between driving ability and participant characteristics, including physical fitness indices, is shown using a correlation table (Table 2). In this study, Pearson’s correlation coefficient calculation method was used. No evidence of association was found between driving ability and any physical fitness factor. Similarly, no evidence of association was found between driving ability and age, BMI, TMT-A, and TMT-B.

### 3.3. Multiple Regression Model Analysis

Multiple regression models were generated to examine the relationship between driving ability and physical fitness factors. Multiple regression analysis was performed using the participants’ age, sex, BMI, TMT-A scores, and the above four items to confirm that multicollinearity did not occur. Similarly, interactions among each factor were also confirmed. A model was then created and analyzed using one selected fitness factor in addition to the aforementioned four items (Table 3). Model 1 was generated using grip strength, an indicator of total body muscle strength, as the physical fitness factor. Similarly, four models were generated using each physical fitness factor: flexibility, open-eye one-leg stand, 10 m obstacle walk, and TUG test. In all models, the coefficient of determination R^2^ showed low values (R^2^ < 0.1). Only grip strength was an important explanatory factor among the physical fitness factors, but the coefficient of determination R^2^ also showed a low value (*p* = 0.01, R^2^ = 0.0608). In addition, the relationship between grip strength and driving ability showed that driving ability scores decreased as grip strength scores increased.

## 4. Discussion

### 4.1. Relationship between Driving Ability and Physical Fitness Factors

The driving ability of community-dwelling older adults was tested by instructing them to drive a car on a public road, and their physical fitness was assessed. In the present study, we analyzed the relationship between driving ability and physical fitness factors using measured data. An analysis adjusted for age, sex, BMI, and cognitive ability revealed only grip strength as an important explanatory factor; however, there was an association between increased grip strength and decreased driving ability. In this study, we aimed to analyze the relationship between driving ability and physical fitness factors among community-dwelling older adults whose driving abilities are expected to decline, clarify the essential physical fitness factors that determine driving ability, and obtain methodological data for more efficient maintenance and improvement of driving ability through healthy exercise practices. However, no valid data were obtained.

The driving ability of older adults has been associated with several factors [39]. A motor vehicle is driven under continuous conditions and is influenced by various factors such as sensory factors, cognitive factors, and physical fitness. In addition, physical function factors influence each other and are associated with driving ability. Anstey et al. reported an association between cognitive and visual functions necessary for safe driving and their influence on driving ability [40]. Doroudgar et al. reported differences in driving patterns depending on the age group, such as older drivers having significantly slower reaction times [41]. Regarding the influence of the external environment, Öztürk et al. conducted a comparative analysis of the differences between daytime and nighttime driving conditions for older and younger drivers [42]. They reported that younger drivers can carry out accurate responses while driving, regardless of lighting conditions, etc., while older drivers are slower in their responses while driving. Karthaus et al. compared young, middle-aged, and older drivers in terms of distraction control and reported that visual and auditory distracting stimuli had a more significant effect on the driving of young and older drivers compared to middle-aged drivers [43]. Therefore, it is necessary to consider different perspectives and factors when discussing driving ability.

Depestele et al. conducted a systematic review that focused on cognitive function and compared the driving performance of older and younger drivers [44]. Their findings echoed the earlier research, highlighting that age-related changes in cognitive function significantly impact driving performance. This study underscores the urgent need for a longitudinal analysis of changes in cognitive function, physical function, and driving ability from younger to older age groups. As physical functions, including cognitive functions, evolve with age, they inevitably influence driving ability. Therefore, it is imperative to study driving ability across all generations, from the young to the middle-aged and then to the older adults, to ensure road safety for all.

Regarding the driving ability of older adults, who are the participants of the present study, various measures for safe driving among older adults exist, as aging-induced changes in physical function directly affect driving ability. Anstey et al. reported that compared with safety education that involved only classroom lectures, education involving driving an actual vehicle reduced the number of severe driving errors among older adults [45]. Hay et al. reported that training older adult drivers using a driving simulator significantly improved driving-related abilities [46]. Freidl et al. reported the possibility of objectively evaluating dangerous driving by recording actual driving situations using smartphones [47]. Regular exercise practice has been reported to maintain and improve driving ability [25].

However, effective interventions are required for older adults to promote healthy aging and prevent caregiving [48]. In many countries, the prevention of frailty in older adults is a critical issue [49]. Frailty control is also specified in the World Health Organization guidelines on “physical activity and sedentary behavior” [50], where regular physical activity, strength training, and multi-component physical activity are recommended for older adults [51]. The importance of physical activity is well known, as numerous studies have shown that it improves the physical capabilities of older adults. Man et al. reported that the pre-frail group was less involved in motor vehicle accidents than the non-frail group [52]. In addition, approaching older drivers before becoming frail is important [52].

Considering these previous studies, we present a perspective that relates healthy exercise practices to the maintenance and improvement of driving ability among older adults. In addition, we suggest that healthy exercise practices are not only for preventing frailty but also for improving the maintenance of driving ability. If healthy exercise and physical activity practices influence the maintenance and improvement of driving ability, we believe that more active and healthy exercise and physical activity will lead to improved health-related quality of life, both indirectly and directly.

Notably, more efficient programs can be proposed by clarifying the physical fitness factors that influence the maintenance and improvement of driving ability. In the present study, we could not clarify the relevant physical fitness. Therefore, the study’s design and research methodology should be examined and refined to clarify the relevant physical fitness factors in the future.

### 4.2. Limitations

First, the cross-sectional analysis in this study only scratched the surface of the complex relationship between driving ability and physical fitness factors among older adults. The data we collected provided a starting point; however, longitudinal studies are required to truly understand the causal relationship and potential for specific physical fitness factors to enhance driving ability. These studies, with their carefully considered designs, are crucial to unlocking the wealth of knowledge in this field.

Second, the drivers included all age groups, from young to older adults. Only the older adults were included in this study, and they were measured. By obtaining data from drivers of all age groups, we believe that it will be possible to analyze the data from multiple perspectives, including the relationship between the data and changes in physical fitness due to aging, and to take an appropriate approach to maintaining safe driving among older adult drivers. If possible, measurement of all age groups should be considered.

Third, the measurement of physical fitness factors in this study was not conducted in a laboratory test in an experimental measurement room but in a field test in a practical format in a gymnasium or similar setting. The measurements were planned and conducted based on the premise to ensure they were taken in ample space and completed quickly, primarily to prevent infection by the new coronavirus. The number of measurement items was limited by space and time, and we had no choice but to reduce the measurement items. To collect more accurate data on physical fitness factors, it is necessary to reconsider the measurement methods and items.

Finally, the ability of older adults to drive a car is greatly influenced by their daily driving conditions, including qualitative and quantitative conditions such as frequency of driving, driving time, and driving distance, as well as environmental factors in their residential area. Older adults living in areas near a suburban core city who continued to drive a car daily were recruited to participate in this study. No detailed conditions were set for daily driving conditions. Therefore, the study participants were from a limited geographic area, and the results do not represent general trends in all areas.

## 5. Conclusions

A single regression analysis revealed that no evidence of association was found between driving ability and any physical fitness factor. An analysis adjusted for age, sex, BMI, and cognitive ability revealed only grip strength as an important explanatory factor. However, increased grip strength was associated with decreased driving ability.

Clarifying the essential physical fitness factors determining driving ability is necessary to develop more efficient programs for maintaining and improving driving ability through healthy exercise practices. We intend to actively conduct further research on this topic.

## Figures and Tables

**Table 1:** Characteristics of subjects.

	All Subjects (*n* = 70)				
	Mean	±	SD	Minimum	Maximum
Female, *n* (%)	54 (77.1%)				
Age (year)	74.9	±	3.8	65	82
Height (cm)	155.1	±	9.1	139.3	185.0
Body weight (kg)	56.3	±	10.5	36.6	90.0
BMI (kg/m^2^)	23.3	±	3.3	17.8	37.5
Grip strength (kg)	23.7	±	5.1	15.4	40.1
Flexibility (cm)	33.6	±	10.1	7.5	57.0
One leg with eye opened balance (seconds)	51.6	±	41.2	1.0	120.0
10 m’ walking with obstacles (seconds)	8.3	±	1.4	6.1	12.2
Five times chair standing up test (seconds)	6.6	±	1.7	4.0	13.5
30 s chair standing up test (times)	21.1	±	4.8	10.0	32.0
Timed Up & Go Test (seconds)	6.3	±	1.0	4.5	9.3
TMT-A:Trail Making Test part A (seconds)	60.1	±	23.2	38.0	176.0
TMT-B:Trail Making Test part B (seconds)	104.5	±	45.2	37.0	300.0
Driving ability Cognition	12.4	±	9.2	0	56
Driving ability Judgment	7.0	±	6.3	0	29
Driving ability Operation	1.4	±	2.7	0	14
Driving ability Cognition + Judgment	19.4	±	10.0	3	57
Driving ability Judgment + Operation	8.4	±	7.0	0	29
Driving ability Cognition + Judgment + Operation	20.8	±	9.8	3	57

BMI: body mass index (kg/m^2^). SD: standard deviation.

**Table 2:** Correlation Coefficients between [Driving ability Judgment + Operation] and each factor.

Variables		1		2		3		4		5		6		7		8		9		10	
1.	Age (year)	-																			
2.	BMI(kg/m^2^)	0.01		-																	
3.	Grip strength (kg)	−0.04		0.11		-															
4.	Flexibility (cm)	−0.04		−0.21		−0.14		-													
5.	One leg with eye opened balance (seconds)	−0.12		−0.21		0.10		0.34	**	-											
6.	10 m’ walking with obstacles (seconds)	0.38	**	0.31	**	−0.29	*	−0.17		−0.40	**	-									
7.	Timed Up & Go Test(S)	0.34	**	0.24	*	−0.18		−0.23		−0.28	*	0.76	**	-							
8.	TMT-A:Trail Making Test part A (seconds)	0.37	**	0.04		−0.10		0.04		−0.13		0.29	*	0.35	**	-					
9.	TMT-B:Trail Making Test part B (seconds)	0.34	**	−0.12		−0.20		0.14		0.00		0.32	**	0.29	*	0.29	*	-			
10.	Driving ability Judgment + Operation	0.15		0.07		0.21		0.07		0.03		0.04		0.15		−0.04		0.02		-	

* *p* < 0.05, ** *p* < 0.01.

**Table 3:** Summary of Regression Model Predictors for Driving Ability.

	β	95% Confidence Interval		*p*		VIF
		Lower Limit	Upper Limit			
**Model 1 (vs. Grip Strength)**						
Age	0.29	0.03	1.03	**0.04**	*	1.32
Sex	−0.32	−10.97	0.33	0.06		2.15
BMI	0.02	−0.45	0.55	0.84		1.03
TMT-A	−0.14	−0.12	0.03	0.28		1.21
**Grip strength**	0.42	0.13	1.04	**0.01**	*	2.04
Adjusted R^2^ = 0.0608						
**Model 2 (vs. Flexibility)**						
Age	0.20	−0.13	0.88	0.14		1.24
Sex	−0.03	−4.79	3.88	0.83		1.16
BMI	0.06	−0.40	0.66	0.62		1.05
TMT-A	−0.12	−0.12	0.04	0.37		1.21
**Flexibility**	−0.05	−0.22	0.14	0.69		1.13
Adjusted R^2^ = 0.0435						
**Model 3 (vs. One leg with Eye Opened Balance)**						
Age	0.20	−0.13	0.88	0.14		1.24
Sex	−0.01	-4.37	4.05	0.94		1.09
BMI	0.08	−0.35	0.71	0.50		1.05
TMT-A	−0.12	−0.12	0.05	0.39		1.22
**One leg with Eye Opened Balance**	0.05	−0.03	0.05	0.67		1.08
Adjusted R^2^ = 0.0431						
**Model 4 (10 m’ walking with obstacles)**						
Age	0.21	−0.14	0.92	0.15		1.38
Sex	−0.02	−4.46	3.95	0.90		1.08
BMI	0.08	−0.37	0.73	0.52		1.12
TMT-A	−0.12	−0.12	0.05	0.39		1.24
**10 m’ walking with obstacles**	−0.04	−1.56	1.22	0.81		1.35
Adjusted R^2^ = 0.0416						
**Model 5 (Time Up & Go Test)**						
Age	0.17	−0.21	0.82	0.24		1.32
Sex	−0.01	−4.30	4.06	0.95		1.08
BMI	0.04	−0.44	0.62	0.73		1.07
TMT-A	−0.15	−0.13	0.04	0.26		1.28
**Timed Up & Go Test**	0.14	−0.94	2.74	0.33		1.29
Adjusted R^2^ = 0.0548						

*β*: standardized regression coefficient, VIF: variance inflation factor. Bold, *: *p* < 0.05.

## Data Availability

The original contribution presented in the study is included in the article material. Further inquiries can be directed to the corresponding author.

## References

[1] Fernandes F., Turra C.M., Rios Neto E.L.G. (2023). World population aging as a function of period demographic conditions. Demogr. Res..

[2] United Nations World Population Prospects—Population Division. https://population.un.org/wpp/.

[3] Mudrazija S., Angel J.L., May J.F., Goldstone J.A. (2022). Population aging and public policy. International Handbook of Population Policies.

[4] Karthaus M., Falkenstein M. (2016). Functional changes and driving performance in older drivers: Assessment and interventions. Geriatrics.

[5] Jeong S.H., Kim E.Y., Lee S.J., Choi W.J., Oh C., Sung H.J., Kim J. (2023). Health status and activity discomfort among elderly drivers: Reality of health awareness. Healthcare.

[6] Mirabet E., Tortosa-Perez M., Tortosa F., González-Sala F. (2023). Evaluation of psychophysical fitness in drivers over 65 years of age. Healthcare.

[7] World Health Organization (2015). World Health Organization Global Status Report on Road Safety 2015.

[8] Park K., Renge K., Nakagawa Y., Yamashita F., Tada M., Kumagai Y. (2021). Aging brains degrade driving safety performances of the healthy elderly. Front. Aging Neurosci..

[9] Hagiya H., Takase R., Honda H., Nakano Y., Otsuka Y., Kataoka H., Uno M., Ueda K., Takahashi M., Ogawa H. (2022). Prevalence of medical factors related to aging among older car drivers: A multicenter, cross-sectional, descriptive study. BMC Geriatr..

[10] Cabinet Office (2020). Traffic Safety White Paper. https://www8.cao.go.jp/koutu/taisaku/r02kou_haku/zenbun/index.html.

[11] Voluntary Return of Driver’s License: Japan National Police Agency Web Site. https://www.npa.go.jp/policies/application/license_renewal/jishuhennou.html.

[12] Road Traffic Law Revised in 2020 (Measures for Elderly Drivers, Review of Eligibility for Second-Class Licenses, etc.) (Effective 13 May 2022): Japan National Police Agency Web Site. http://www.npa.go.jp/bureau/traffic/r2kaisei_main.html.

[13] Dugan E., Lee C.M. (2013). Biopsychosocial Risk Factors for Driving Cessation: Findings from the Health and Retirement Study. https://journals.sagepub.com/doi/abs/10.1177/0898264313503493.

[14] Edwards J.D., Lunsman M., Perkins M., Rebok G.W., Roth D.L. (2009). Driving cessation and health trajectories in older adults. J. Gerontol. S A.

[15] Chihuri S., Mielenz T.J., DiMaggio C.J., Betz M.E., DiGuiseppi C., Jones V.C., Li G. (2016). Driving cessation and health outcomes in older adults. J. Am. Geriatr. Soc..

[16] Tang K.F., Teh P.-L., Lim W.M., Lee S.W.H. (2022). Perspectives on mobility among older adults living with different frailty and cognitive statuses. J. Transp. Health.

[17] Che Had N.H., Alavi K., Md Akhir N., Muhammad Nur I.R., Shuhaimi M.S.Z., Foong H.F. (2023). A scoping review of the factor associated with older adults’ mobility barriers. Int. J. Env. Res. Public Health.

[18] Jalenques I., Rondepierre F., Rachez C., Lauron S., Guiguet-Auclair C. (2020). Health-related quality of life among community-dwelling people aged 80 years and over: A cross-sectional study in France. Health Qual. Life Outcomes.

[19] Hajek A., Brettschneider C., Lühmann D., van den Bussche H., Wiese B., Mamone S., Weyerer S., Werle J., Leve V., Fuchs A. (2021). Driving status and health-related quality of life among the oldest old: A population-based examination using data from the AgeCoDe–AgeQualiDe prospective cohort study. Aging Clin. Exp. Res..

[20] Cirella G.T., Bąk M., Kozlak A., Pawłowska B., Borkowski P. (2019). Transport innovations for elderly people. Res. Transp. Bus. Manag..

[21] Lajunen T., Sullman M.J.M. (2021). Attitudes toward four levels of self-driving technology among elderly drivers. Front. Psychol..

[22] Oxley J., Logan D.B., Coxon S., Koppel S. (2022). Understanding current and future transport needs of older Australian drivers to guide development of sustainable and smart initiatives to support safe mobility of older adults. Sustainability.

[23] Kosuru V.S.R., Venkitaraman A.K., Kosuru V.S.R., Venkitaraman A.K. (2023). Advancements and challenges in achieving fully autonomous self-driving vehicles. World J. Adv. Res. Rev..

[24] Marottoli R.A., Allore H., Araujo K.L.B., Iannone L.P., Acampora D., Gottschalk M., Charpentier P., Kasl S., Peduzzi P. (2007). A randomized trial of a physical conditioning program to enhance the driving performance of older persons. J. Gen. Intern. Med..

[25] Katayama A., Hase A., Miyatake N. (2023). Enhancing driving ability in older adults through health exercises and physical activity: A randomized controlled trial. Int. J. Envrion. Res. Public. Health.

[26] Kitaoka H., Kurahashi T., Mori H., Iwase T., Machida T., Kozato A., Yamashita M., Kisanuki Y. (2009). A development of a traffic simulator for safety evaluation—Reproduction of traffic accidents and evaluation of safety systems. Rev. Automot. Eng..

[27] Casutt G., Martin M., Jäncke L. (2016). Driving simulator training is associated with reduced inhibitory workload in older drivers. Geriatrics.

[28] Ministry of Education, Culture, Sports, Science, and Technology New Physical Fitness Test Guidelines. https://www.mext.go.jp/a_menu/sports/stamina/03040901.htm.

[29] Sports Agency New Fitness Test Guideline. https://www.mext.go.jp/sports/b_menu/sports/mcatetop03/list/detail/1408001.htm.

[30] Kawabata Y., Hiura M. (2008). The CS−30 test is a useful assessment tool for predicting falls in community-dwelling elderly people. Rigakuryoho Kagaku.

[31] Nakazono T., Kamide N., Ando M. (2014). The reference values for the chair stand test in healthy Japanese older people: Determination by meta-analysis. J. Phys. Ther. Sci..

[32] Rikli R.E., Jones C.J. (1999). Development and validation of a functional fitness test for community-residing older adults. J. Aging Phys. Act..

[33] Tiedemann A., Shimada H., Sherrington C., Murray S., Lord S. (2008). The comparative ability of eight functional mobility tests for predicting falls in community-dwelling older people. Age Ageing.

[34] Shumway-Cook A., Brauer S., Woollacott M. (2000). Predicting the probability for falls in community-dwelling older adults using the timed UP & go test. Phys. Ther..

[35] Sprint G., Cook D.J., Weeks D.L. (2015). Toward automating clinical assessments: A survey of the timed UP and Go. IEEE Rev. Biomed. Eng..

[36] Giovagnoli A.R., Del Pesce M., Mascheroni S., Simoncelli M., Laiacona M., Capitani E. (1996). Trail making test: Normative values from 287 normal adult controls. Ital. J. Neurol. Sci..

[37] Tombaugh T.N. (2004). Trail making Test A and B: Normative data stratified by age and education. Arch. Clin. Neuropsychol..

[38] Bowie C.R., Harvey P.D. (2006). Administration and interpretation of the trail making test. Nat. Protoc..

[39] Anstey K.J., Wood J., Lord S., Walker J.G. (2005). Cognitive, sensory and physical factors enabling driving safety in older adults. Clin. Psychol. Rev..

[40] Anstey K.J., Horswill M.S., Wood J.M., Hatherly C. (2012). The role of cognitive and visual abilities as predictors in the multifactorial model of driving safety. Accid. Anal. Prev..

[41] Doroudgar S., Chuang H.M., Perry P.J., Thomas K., Bohnert K., Canedo J. (2017). Driving Performance Comparing Older versus Younger Drivers. Traffic Inj. Prev..

[42] Öztürk İ., Merat N., Rowe R., Fotios S. (2023). The Effect of Cognitive Load on Detection-Response Task (DRT) Performance during Day- and Night-Time Driving: A Driving Simulator Study with Young and Older Drivers. Transp. Res. Part F Traffic Psychol. Behav..

[43] Karthaus M., Wascher E., Falkenstein M., Getzmann S. (2020). The Ability of Young, Middle-Aged and Older Drivers to Inhibit Visual and Auditory Distraction in a Driving Simulator Task. Transp. Res. Part F Traffic Psychol. Behav..

[44] Depestele S., Ross V., Verstraelen S., Brijs K., Brijs T., van Dun K., Meesen R. (2020). The Impact of Cognitive Functioning on Driving Performance of Older Persons in Comparison to Younger Age Groups: A Systematic Review. Transp. Res. Part F Traffic Psychol. Behav..

[45] Anstey K.J., Eramudugolla R., Kiely K.M., Price J. (2018). Effect of tailored on-road driving lessons on driving safety in older adults: A randomised controlled trial. Accid. Anal. Prev..

[46] Hay M., Adam N., Bocca M.-L., Gabaude C. (2016). Effectiveness of two cognitive training programs on the performance of older drivers with a cognitive self-assessment bias. Eur. Transp. Res. Rev..

[47] Freidlin R.Z., Dave A.D., Espey B.G., Stanley S.T., Garmendia M.A., Pursley R., Ehsani J.P., Simons-Morton B.G., Pohida T.J. (2018). Measuring risky driving behavior using an mhealth smartphone app: Development and evaluation of gForce. JMIR Mhealth Uhealth.

[48] Jadczak A.D., Makwana N., Luscombe-Marsh N., Visvanathan R., Schultz T.J. (2018). Effectiveness of exercise interventions on physical function in community-dwelling frail older people: An umbrella review of systematic reviews. JBI Database Syst. Rev. Implement. Rep..

[49] Xue Q.L. (2011). The frailty syndrome: Definition and natural history. Clin. Geriatr. Med..

[50] WHO Guidelines on Physical Activity and Sedentary Behaviour. https://www.who.int/publications-detail-redirect/9789240015128.

[51] Li Y., Gao Y., Hu S., Chen H., Zhang M., Yang Y., Liu Y. (2023). Effects of multicomponent exercise on the muscle strength, muscle endurance and balance of frail older adults: A meta-analysis of randomised controlled trials. J. Clin. Nurs..

[52] Man C., Ng L.S., Molnar L.J., Eby D.W., Ryan L.H., DiGuiseppi C., Strogatz D., Betz M.E., Hill L., Guralnik J. (2019). Frailty phenotype and self-reported crashes and driving space: Baseline AAA LongROAD. J. Transp. Health.

